# Development and Validation of a Prediction Model for Respiratory Failure in Patients With Sepsis-Associated Acute Kidney Injury (SA-AKI) Within 48 Hours of Admission

**DOI:** 10.1155/emmi/5517872

**Published:** 2025-10-17

**Authors:** Bin Wang, Fengxiang Zhang

**Affiliations:** ^1^Department of Critical Care Medicine, The First Affiliated Hospital of Jinzhou Medical University, Jinzhou, Liaoning 121000, China; ^2^Department of Emergency, DongYang People's Hospital, Dongyang, Zhejiang, China

**Keywords:** acute kidney injury, invasive ventilation, machine learning, prediction model, sepsis

## Abstract

**Objective:**

To identify patients with sepsis-associated acute kidney injury (SA-AKI) at high risk of respiratory failure within 48 h of admission and enable timely intervention to improve patient prognosis.

**Methods:**

Data from SA-AKI patients admitted to Dongyang People's Hospital between June 2012 and October 2024 were collected, including gender, age, and blood biochemical indicators at admission. Patients were randomly divided into training and validation groups. Independent risk factors for respiratory failure were identified in the training group, and a nomogram prediction model was developed. The model's discriminative ability was assessed using the area under the receiver operating characteristic (ROC) curve (AUC), and its calibration was evaluated using the GiViTi calibration plot. Clinical effectiveness was examined using decision curve analysis (DCA). Cross-validation was performed to test the model's stability using kappa value. The model was subsequently validated in the validation group. Sequential Organ Failure Assessment (SOFA)-based, National Early Warning Score (NEWS)-based, and various other machine learning models were also established and compared with the proposed model using DeLong's test after Bonferroni correction.

**Results:**

A total of 702 patients were included in the study. Independent risk factors for respiratory failure included D-dimer, lactate, pro-BNP, albumin, globulin, transcutaneous blood oxygen saturation, and pulmonary infection. The AUC values for the training and validation groups were 0.818 and 0.795, respectively, with calibration plot *p* values of 0.973 and 0.864. The DCA curves for both groups indicated superior clinical utility compared with extreme scenarios. This model owned a kappa value of 0.628, indicating for good stability. The SOFA model achieved AUC values of 0.583 (training group) and 0.763 (validation group), while the NEWS model had AUC values of 0.628 (training) and 0.618 (validation). DeLong's test confirmed that the proposed model outperformed SOFA and NEWS models (*p* < 0.05). In the validation group, the AUC values for SVM, C5.0, XGBoost, and integrated models were 0.781, 0.757, 0.759, and 0.778, respectively, with comparable discriminative ability to the nomogram (*p* > 0.05).

**Conclusion:**

The nomogram developed in this study based on D-dimer, lactate, pro-BNP, albumin, globulin, transcutaneous blood oxygen saturation, and pulmonary infection was found to effectively predict respiratory failure risk in SA-AKI patients within 48 h of admission.

## 1. Introduction

Sepsis is a life-threatening syndrome of organ dysfunction caused by an inappropriate host immune response to infection [1, 2]. A common complication of sepsis is acute kidney injury (AKI), referred to as sepsis-associated (SA)-AKI, which significantly worsens patient survival [3, 4]. According to KDIGO guidelines, SA-AKI is defined as AKI occurring within a week of sepsis onset, as proposed by the Acute Disease Quality Initiative (ADQI) group in 2023 [5]. SA-AKI is further classified into early SA-AKI (occurring within 48 h of sepsis onset) and late SA-AKI (occurring between 48 h and 7 days after sepsis onset). Compared to sepsis or AKI alone, SA-AKI is associated with a poorer prognosis [3, 6], increasing in-hospital mortality rates by six- to eightfold [7].

Respiratory failure, a frequent complication of sepsis, is a strong independent risk factor for increased mortality in these patients [8]. Acute respiratory distress syndrome (ARDS) is the most common manifestation [9], although other pulmonary impairments, such as hypoxemia, also contribute to worse outcomes, including death. Sepsis, AKI, and respiratory failure are closely interrelated, each influencing the progression and outcomes of the others [10–12]. In SA-AKI patients, the early onset of respiratory failure is a critical predictor of poor prognosis [13]. Therefore, developing a model to predict respiratory failure in SA-AKI patients within 1 month of hospitalization is essential to improve outcomes.

Currently, traditional models, such as the Sequential Organ Failure Assessment (SOFA) [14], Acute Physiology and Chronic Health Evaluation II (APACHE II) [15], and National Early Warning Score (NEWS) [16], are commonly used in clinical practice to predict the risk of organ dysfunction and prognosis in sepsis patients. However, the accuracy of these scores in predicting the risk of respiratory failure in sepsis patients is questionable. Advances in computational tools have led to new models, primarily targeting ARDS prediction in sepsis patients [17–19]. While some models address respiratory failure risk [20, 21], they are aimed at all sepsis patients, and their suitability for SA-AKI patients remains unclear.

To address this gap, a predictive model for respiratory failure risk in SA-AKI patients within 48 h of admission is needed. Such a model could stratify patients by risk level, enhance physician–patient communication, and enable early interventions to reduce mortality in high-risk patients.

## 2. Materials and Methods

### 2.1. Study Subjects

Sepsis patients admitted to Dongyang People's Hospital between June 2012 and October 2024 were included in this study. Inclusion criteria were (1) meeting the Sepsis-3.0 diagnostic criteria (infection with a SOFA score increase of ≥ 2) and (2) meeting AKI criteria (creatinine increase > 26.5 μmol/L within 48 h, a 50% increase in baseline creatinine, or urine output < 0.5 mL/kg/h for over 6 h). Exclusion criteria included (1) age under 18 years, (2) unknown baseline renal function, (3) refusal of therapy, or (4) patients who required urgent abdominal surgery.

### 2.2. Observation Indicators

Data collected included patients' age, gender, and medical history, such as diabetes, hypertension, cerebral infarction, chronic liver disease (cirrhosis), chronic lung diseases (e.g., chronic obstructive pulmonary disease, interstitial pneumonia, and chronic pulmonary fibrosis), chronic kidney disease (chronic renal insufficiency and nephrotic syndrome), chronic heart disease (NYHA class II or higher), malignant tumors, and leukemia. Blood parameters at admission included high-sensitivity C-reactive protein, B-type natriuretic peptide, total bilirubin, aspartate aminotransferase, cholinesterase, triglycerides, lactate, creatinine, D-dimer, magnesium, sodium, potassium, calcium, prothrombin time, white blood cell count, platelet count, hemoglobin, globulin, and albumin. Vital signs at admission comprised mean arterial pressure, transcutaneous oxygen saturation, heart rate, body temperature, respiratory rate, and Glasgow Coma Scale (GCS) score. Infection sites included gastrointestinal, pulmonary, intracranial, biliary, and urinary tract infections. Respiratory failure was defined as an oxygenation index below 300.

### 2.3. Ethical Oversight

The study adhered to the principles of the Declaration of Helsinki and its amendments and was approved by the Ethics Committee of Dongyang People's Hospital (approval number: Dong Ren Yi 2024-YX-096). Written informed consent was obtained from all patients and their families. Data were anonymized and sourced from the Le Jiu database using a coded numbering system.

### 2.4. Variable Selection

Variables with over 20% missing data were excluded after an initial data review. Missing data for the remaining variables were imputed using the mice package for multiple imputations. In detail, five datasets were generated by using the predictive mean matching method and the optimal one was obtained by “with” function and “pool” function. Imputed data was extracted by “complete” function. Based on clinical norms, some continuous variables were transformed into categorical variables. The dataset was then randomly divided into training and validation groups at a 7:3 ratio. Univariate analysis was performed in the training group, and variables with significance at *p* < 0.01 were subjected to multicollinearity assessment using variance inflation factors (VIFs). Variables with a VIF < 10 were considered free from significant multicollinearity. The boxTidwell function was used to assess linearity between variables and the logit of probability (logitp), with *p* < 0.05 indicating nonlinearity. Multivariate logistic regression was then conducted, followed by stepwise regression to identify independent risk factors and construct a predictive nomogram.

### 2.5. Model Evaluation and Validation

The model's discrimination was evaluated using the area under the receiver operating characteristic (ROC) curve (AUC), with AUC > 0.75 indicating strong discrimination [22]. Calibration was assessed using calibration plots, with Hosmer–Lemeshow *p* > 0.05 indicating acceptable calibration [23]. Clinical effectiveness was evaluated using decision curve analysis (DCA), where the model's curve exceeding the extremes (all or none) demonstrated superior effectiveness [24]. Validation of the model was repeated in the validation group. The stability of the established model was evaluated by 10-fold cross validation using the kappa value [25].

### 2.6. Model Comparisons

The performance of the nomogram was compared with that of the SOFA and NEWS scoring models using DeLong's test and an adjusted *p* value was obtained after Bonferroni correction. Additionally, three machine learning models—XGBoost, C5.0, and SVM—were constructed in the validation group, and an ensemble model using stacking [26] was developed for comparison with the nomogram.

### 2.7. Statistical Analyses

Variables that were normally distributed were expressed as the mean ± standard deviation, and group differences were analyzed using *t*-tests. Non-normally distributed variables were expressed as medians and interquartile ranges and analyzed using the Mann–Whitney *U* test. Categorical variables were expressed as percentages and analyzed using the chi-square test. All statistical analyses were conducted using R.

## 3. Results

### 3.1. Baseline Comparisons of the Training and Validation Groups

A total of 702 SA-AKI patients were included in this study (426 males and 276 females). The training group consisted of 492 patients, and the validation group included 210 patients. There were no statistically significant differences between the two groups for any of the analyzed variables, as shown in Table 1.

### 3.2. Univariate Analysis Results

Univariate analysis in the training group identified lactate, D-dimer, B-type natriuretic peptide precursor (pro-BNP), prothrombin time, albumin, globulin, transcutaneous oxygen saturation, respiratory rate, and pulmonary infection as significant variables (*p* < 0.001, Table 2). The VIF values for these variables were 1.270267, 1.111818, 1.060794, 1.108886, 1.096772, 1.071812, 1.083081, 1.047423, and 1.077263, respectively. The VIF values for all variables were less than 5, indicating no multicollinearity among variables. The *p* values for linearity between these continuous variables and logitp were 0.2129, 0.5007, 0.2966, 0.3158, 0.8808, 0.9077, 0.9935, and 0.8020, respectively, all of which were greater than 0.05, confirming a linear relationship.

### 3.3. Multivariate and Stepwise Regression Analysis Results

Multivariate and stepwise regression analyses confirmed seven significant predictors: lactate, D-dimer, pro-BNP, albumin, globulin, transcutaneous oxygen saturation, and pulmonary infection (*p* < 0.05), as shown in Table 3.

### 3.4. Nomogram Development

**Figure 1:**
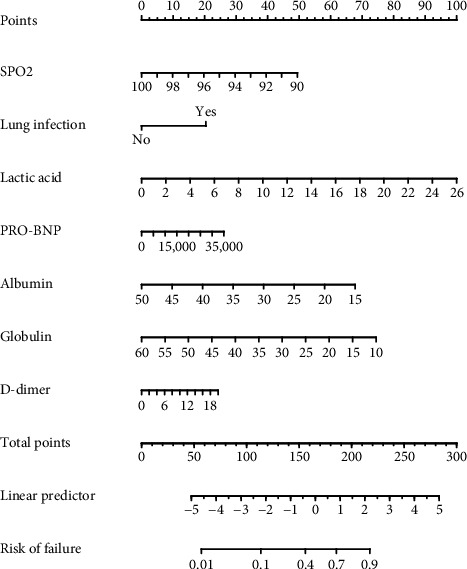
Risk-prediction nomogram for respiratory failure within 48 h following admission in patients with sepsis.

A nomogram was developed based on the identified independent risk factors (Figure 1). Each factor was assigned a score, with the total score used to estimate the probability of respiratory failure.

### 3.5. Assessment of Model Discrimination, Calibration, and Clinical Efficacy

**Figure 2:**
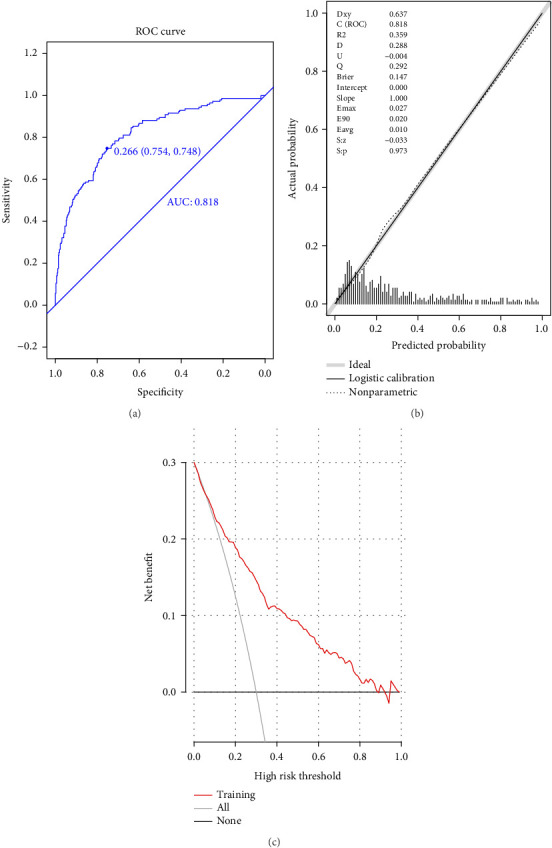
Evaluation of prediction model in modeling group. (a) ROC curves; (b) calibration chart; and (c) DCA curves.

The ROC curve (Figure 2(a)) showed an AUC of 0.818 (95% CI: 0.777–0.860), with a cutoff value of 0.266, specificity of 75.4% (95% CI: 70.8%–80.0%), sensitivity of 74.8% (95% CI: 67.1%–81.8%), accuracy of 75.2%, positive predictive value of 55.4% (95% CI: 48.4%–62.5%), and negative predictive value of 88.0% (95% CI: 84.3%–91.6%). The calibration plot (Figure 2(b)) yielded a *p* value of 0.973, indicating excellent calibration. DCA (Figure 2(c)) showed that the model's curve surpassed the extreme curves, confirming satisfactory discrimination, calibration, and clinical utility.

### 3.6. Model Validation

**Figure 3:**
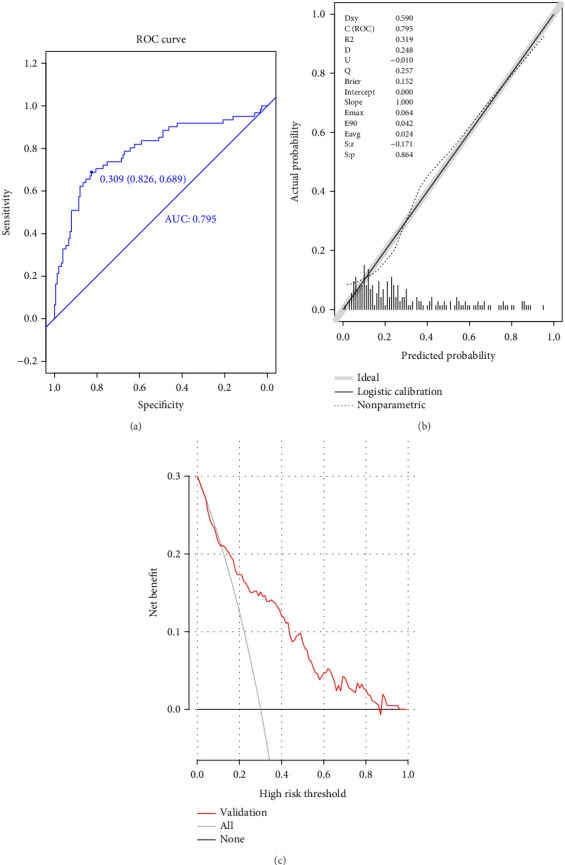
Evaluation of prediction model in validation group. (a) ROC curves; (b) calibration chart; and (c) DCA curves.

The ROC curve (Figure 3(a)) demonstrated an AUC of 0.795 (95% CI: 0.723–0.860), with a model cutoff value of 0.309. The model achieved a specificity of 82.6% (95% CI: 76.5%–88.6%), sensitivity of 68.9% (95% CI: 57.4%–80.3%), accuracy of 78.6% (78.4%–78.7%), positive predictive value of 61.8% (95% CI: 50.2%–73.3%), and negative predictive value of 86.6% (95% CI: 81.0%–92.2%). The calibration plot (Figure 3(b)) yielded a *p* value of 0.864, indicating good calibration. The DCA curve (Figure 3(c)) confirmed strong clinical effectiveness, with the model's curve positioned above the extreme curves, underscoring its robust discrimination, calibration, and overall clinical utility. Our model owned a kappa value of 0.628 in cross validation, suggesting a good stability.

### 3.7. Comparison With the SOFA and NEWS Models

**Figure 4:**
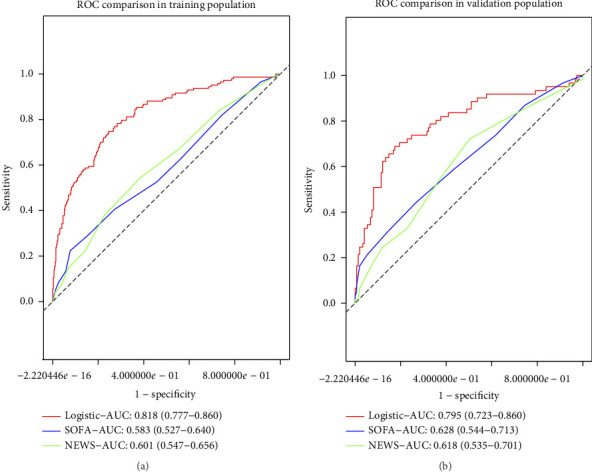
Comparison of ROCs for models. (a) Comparison to the models based on SOFA and NEWS scoring systems in training population and (b) comparison to the models based on SOFA and NEWS scoring systems in the validation group.

In the training group, the AUC values for the SOFA and NEWS models were 0.583 (95% CI: 0.527–0.640) and 0.601 (95% CI: 0.547–0.656), respectively, as shown in Figure 4(a). In the validation group, the AUC values were 0.628 (95% CI: 0.544–0.713) for SOFA and 0.618 (95% CI: 0.535–0.701) for NEWS, as shown in Figure 4(b). DeLong's test results for both groups revealed *p* < 0.001, demonstrating that our model significantly outperformed the SOFA and NEWS models in terms of discrimination.

### 3.8. Comparison With Other Machine Learning Models

**Figure 5:**
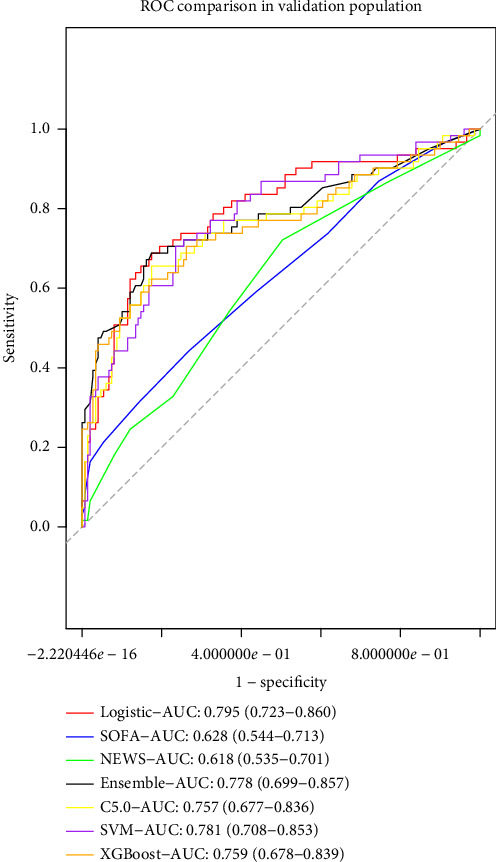
Comparison of ROCs for all models.

In the validation group, the comparison between our model and other machine learning models is presented in Figure 5. DeLong's test results for the ensemble, SVM, C5.0, and XGBoost models yielded *p* values of 0.754, 0.778, 0.482, and 0.2, respectively, indicating no statistically significant differences between these models and ours (Table 4). The detailed information for other metrics including prediction accuracy, negative predictive value, sensitivity, and specificity were also provided in Table 4.

## 4. Discussion

This retrospective study of SA-AKI patients admitted to the hospital over the last several years identified several independent risk factors for respiratory failure within 48 h of admission, including lactate, D-dimer, pro-BNP, albumin, globulin, transcutaneous oxygen saturation, and pulmonary infection. Of these, albumin and globulin were established as protective factors. The predictive model we developed based on these variables demonstrated strong performance.

Lactate, a marker of oxygen metabolism, is closely linked to respiratory and circulatory function. Previous studies have shown that lactate levels correlate with sepsis prognosis and are directly associated with the onset of respiratory failure in septic patients [20, 27]. D-dimer, a marker of thrombosis, is elevated in sepsis due to abnormal coagulation. Thrombosis in the lungs, whether microvascular or macrovascular, disrupts ventilation–perfusion ratios, contributing to respiratory failure [28]. Albumin, an indicator of nutritional status, affects respiratory muscle function and plasma oncotic pressure. Low albumin levels can lead to pulmonary edema and respiratory failure [20, 29, 30]. Globulin, an acute-phase protein involved in the immune-inflammatory response, rises during pathogen invasion or in response to toxin exposure [31]. A decrease in globulin may indicate poor immune function, worsening sepsis progression and respiratory failure [32]. Pro-BNP reflects cardiac function, with elevated levels indicating ventricular tension and being linked to poor cardiac function. Sepsis itself can cause an increase in pro-BNP levels, and fluid resuscitation during sepsis treatment can also lead to pro-BNP elevation. As cardiac function deteriorates, respiratory failure is increasingly likely to occur [33, 34]. In the absence of poisoning, transcutaneous oxygen saturation reflects oxygen partial pressure [35]. Low levels at admission suggest impaired respiratory function due to sepsis, increasing the risk of respiratory failure. Pulmonary infections cause alveolar and mucosal damage, leading to immune dysfunction, secondary infections, and respiratory failure [36, 37].

Although most enrolled variables could be obtained in clinical practice, some ones have not routinely tested in source-limited regions, such as pro-BNP. Fortunately, our model owned an AUC of after excluding pro-BNP, indicating for good prediction performance. This phenomenon could be explained by the main contributors of other variable (including lactate acid, albumin, globulin, and SaO_2_), further broadening the applicability in clinical settings. Because the enrolled cases in this study were not merely from ICU or general wards, we believe that this established model would perform well when the enrolled variables were available.

When predicting the risk of respiratory failure in SA-AKI patients within 48 h of admission, our model demonstrated superior discrimination compared to the traditional SOFA [14] and NEWS [16] models, which were developed before the emergence of certain biomarkers, such as lactate and D-dimer. These newer biomarkers, now widely used in modern clinical settings, have improved the accuracy of our model. While testing these biomarkers incurs additional costs, the benefits of early prediction and intervention in SA-AKI patients outweigh the expenses. Furthermore, traditional scoring systems do not have an effective ability for predicting respiratory failure as they were not originally established specially for SA-AKI patients. By integrating our model into electronic medical record systems, the risk of respiratory failure among SA-AKI patients could be predicted as they are admitted to hospital based on enrolled variables that could be automatically extracted from laboratory examinations. Once patients with high risk of respiratory failure are identified, more active interventions could be taken by medica staffs to improve the final prognosis.

In the validation group, our model performed comparably to other machine learning models, including C5.0, XGBoost, SVM, and ensemble methods. The absence of significant differences suggests that our model effectively captures the essential features of the data. Unlike machine learning models, which often rely on numerous variables and can be less interpretable, our model offers higher clarity and practical applicability by using less variables and being displayed in a visualized format. Machine learning models are generally preferred only when their predictive advantage is substantial [38].

Nevertheless, our study has limitations. It is a single-center study, requiring external validation or multicenters to confirm the model's accuracy. Additionally, as a retrospective study, it is susceptible to selection bias. Due to potential unavailability of certain enrolled variables in source-limited regions, this established model may not perform as effectively in general wards as it does in our study.

## 5. Conclusion

In summary, we herein developed a nomogram that can accurately predict the risk of respiratory failure in SA-AKI patients within 48 h of admission. This model includes seven indicators and can assist clinicians in the decision-making, management, and treatment of SA-AKI patients.

## Figures and Tables

**Table 1:** Baseline characteristics of the modeling population and validation population^ab^.

Variables	Total (*n* = 702)	Training (*n* = 492)	Testing (*n* = 210)	*p*
Gender				0.857
Male	426 (61)	297 (60)	129 (61)	
Female	276 (39)	195 (40)	81 (39)	
Age (years)	73 (60, 82)	72 (60, 82)	74 (61, 82)	0.461
HS-CRP (mg/L)	144.55 (76.12, 200)	144.7 (77.64, 200)	143.19 (72.5, 200)	0.703
Alanine transaminase (U/L)	23 (14, 42)	24 (14, 43)	20 (13, 38)	0.053
Triglyceride (mmol/L)	2.95 (2.41, 3.6)	2.92 (2.37, 3.56)	3.04 (2.55, 3.7)	0.113
Total bilirubin (umol/L)	11.75 (7.5, 20.9)	11.9 (7.68, 21.1)	11.25 (7.1, 20.38)	0.251
Creatinine (umol/L)	184 (148, 269)	183 (149, 256.5)	191 (147, 303.5)	0.135
Lactic acid (mmol/L)	2.2 (1.4, 3.8)	2.3 (1.4, 3.82)	2.1 (1.3, 3.8)	0.256
PRO-BNP (pg/mL)	2662.5 (938.4, 8183)	2509 (936.62, 7850.75)	2885.5 (968.78, 8576.25)	0.427
Cholinesterase (U/L)	4063 (3045.25, 5085)	4008 (3023, 5081.25)	4169 (3150.5, 5145.25)	0.178
Prothrombin time (s)	15.5 (14.3, 16.9)	15.5 (14.3, 16.9)	15.4 (14.1, 17.17)	0.777
D-dimer (mg/L)	3.75 (2.1, 8.09)	3.62 (2.09, 7.61)	3.97 (2.2, 8.58)	0.558
Potassium (mmol/L)				0.82
3.5–5.5	449 (64)	318 (65)	131 (62)	
< 3.5	209 (30)	143 (29)	66 (31)	
> 5.5	44 (6)	31 (6)	13 (6)	
Sodium (mmol/L)				0.41
135–145	334 (48)	226 (46)	108 (51)	
< 135	332 (47)	240 (49)	92 (44)	
> 145	36 (5)	26 (5)	10 (5)	
Magnesium (mmol/L)				1
0.75–1.25	452 (64)	316 (64)	136 (65)	
< 0.75	245 (35)	172 (35)	73 (35)	
> 1.25	5 (1)	4 (1)	1 (0)	
Calcium (mmol/L)				0.639
2.25–2.75	43 (6)	29 (6)	14 (7)	
< 2.25	656 (93)	460 (93)	196 (93)	
> 2.25	3 (0)	3 (1)	0 (0)	
White blood cell (∗10^−9^/L)				0.399
4–10	211 (30)	152 (31)	59 (28)	
< 4	54 (8)	41 (8)	13 (6)	
> 10	437 (62)	299 (61)	138 (66)	
Hemoglobin (∗10^−9^/L)				0.879
110–160	365 (52)	256 (52)	109 (52)	
< 110	317 (45)	223 (45)	94 (45)	
> 160	20 (3)	13 (3)	7 (3)	
Platelet (∗10^−9^/L)				0.739
100–300	465 (66)	330 (67)	135 (64)	
< 100	203 (29)	138 (28)	65 (31)	
> 300	34 (5)	24 (5)	10 (5)	
Albumin (g/L)	29 (25.72, 31.8)	28.6 (25.6, 31.63)	29.65 (26.1, 32.27)	0.124
Globulin (g/L)	26.6 (23.5, 30.4)	26.6 (23.48, 30.52)	26.55 (23.72, 30.17)	0.876
SPO_2_ (%)	97 (95, 98)	97 (95, 98.25)	97 (95, 98)	0.581
Temperature (°C)				0.53
36–37.5	356 (51)	256 (52)	100 (48)	
< 36	41 (6)	27 (5)	14 (7)	
> 37.5	305 (43)	209 (42)	96 (46)	
MAP (mmHg)				0.447
70–105	423 (60)	289 (59)	134 (64)	
< 70	196 (28)	142 (29)	54 (26)	
> 105	83 (12)	61 (12)	22 (10)	
Heart rate (times/min)	98 (85.25, 115)	99 (86, 117)	96 (83.25, 113)	0.136
Breathe rate (times/min)	20 (18, 22)	20 (18, 22)	20 (18, 22)	0.611
GCS	15 (15, 15)	15 (15, 15)	15 (15, 15)	0.23
Diabetes				0.446
No	554 (79)	384 (78)	170 (81)	
Yes	148 (21)	108 (22)	40 (19)	
Hypertension				0.805
No	351 (50)	248 (50)	103 (49)	
Yes	351 (50)	244 (50)	107 (51)	
Cerebral infarction				0.899
No	668 (95)	469 (95)	199 (95)	
Yes	34 (5)	23 (5)	11 (5)	
Cancer				0.981
No	593 (84)	415 (84)	178 (85)	
Yes	109 (16)	77 (16)	32 (15)	
Chronic lung disease				0.45
No	673 (96)	474 (96)	199 (95)	
Yes	29 (4)	18 (4)	11 (5)	
Chronic heart disease				0.86
No	679 (97)	475 (97)	204 (97)	
Yes	23 (3)	17 (3)	6 (3)	
Chronic liver disease				0.368
No	673 (96)	469 (95)	204 (97)	
Yes	29 (4)	23 (5)	6 (3)	
Chronic kidney disease				0.559
No	637 (91)	449 (91)	188 (90)	
Yes	65 (9)	43 (9)	22 (10)	
Leukemia				0.433
No	695 (99)	488 (99)	207 (99)	
Yes	7 (1)	4 (1)	3 (1)	
Intracranial infection				1
No	700 (100)	490 (100)	210 (100)	
Yes	2 (0)	2 (0)	0 (0)	
Lung infection				0.461
No	503 (72)	348 (71)	155 (74)	
Yes	199 (28)	144 (29)	55 (26)	
Biliary infection				0.431
No	652 (93)	454 (92)	198 (94)	
Yes	50 (7)	38 (8)	12 (6)	
Urinary infection				0.902
No	568 (81)	397 (81)	171 (81)	
Yes	134 (19)	95 (19)	39 (19)	
Gastrointestinal infection				0.902
No	568 (81)	397 (81)	171 (81)	
Yes	134 (19)	95 (19)	39 (19)	
NEWS score	4 (3, 6)	4 (3, 6)	4 (3, 6)	0.989
SOFA score	5 (4, 7)	5 (4, 7)	5 (4, 7)	0.622

*Note:* SPO_2_, pulse oxygen saturation.

Abbreviations: GCS, Glasgow coma score; HS-CRP, high-sensitivity C-reactive protein; MAP, mean arterial pressure

^a^Continuous variables are described as means and quarterbacks due to non-normal distribution. Categories varies are analyzed by the *χ*^2^ test and continuous variables are analyzed by the Wilcoxon rank sum test.

^b^First examination index following admission.

**Table 2:** Univariate analysis between respiratory failure and no respiratory failure in training population^ab^.

Variables	Total (*n* = 492)	Control group (*n* = 349)	Respiratory failure (*n* = 143)	*p*
Gender				0.397
Male	297 (60)	206 (59)	91 (64)	
Female	195 (40)	143 (41)	52 (36)	
Age (years)	72 (60, 82)	71 (60, 81)	75 (63, 83)	0.061
HS-CRP (mg/L)	144.7 (77.64, 200)	148.2 (83.35, 200)	140 (58.38, 200)	0.186
Alanine transaminase (U/L)	24 (14, 43)	24 (14, 39)	26 (16, 61)	0.059
Triglyceride (mmol/L)	2.92 (2.37, 3.56)	2.97 (2.46, 3.63)	2.72 (2.12, 3.34)	0.005
Total bilirubin (umol/L)	11.9 (7.68, 21.1)	11 (7.5, 18.6)	14.2 (8.25, 29.8)	0.001
Creatinine (umol/L)	183 (149, 256.5)	183 (149, 262)	182 (145, 248.5)	0.518
Lactic acid (mmol/L)	2.3 (1.4, 3.82)	2 (1.3, 3.2)	3.6 (2.05, 6.75)	< 0.001
PRO-BNP (pg/mL)	2509 (936.62, 7850.75)	2032 (836.7, 5887)	5005.5 (1510, 16289.5)	< 0.001
Cholinesterase (U/L)	4008 (3023, 5081.25)	4095 (3076, 5160)	3719 (2835.5, 4769.5)	0.01
Prothrombin time (s)	15.5 (14.3, 16.9)	15.3 (14.2, 16.6)	15.9 (14.6, 17.9)	< 0.001
D-dimer (mg/L)	3.62 (2.09, 7.61)	3.18 (1.88, 6.53)	5.17 (2.69, 12.7)	< 0.001
Potassium (mmol/L)				0.394
3.5–5.5	318 (65)	222 (64)	96 (67)	
< 3.5	143 (29)	107 (31)	36 (25)	
> 5.5	31 (6)	20 (6)	11 (8)	
Sodium (mmol/L)				0.014
135–145	226 (46)	155 (44)	71 (50)	
< 135	240 (49)	181 (52)	59 (41)	
> 145	26 (5)	13 (4)	13 (9)	
Magnesium (mmol/L)				0.96
0.75–1.25	316 (64)	223 (64)	93 (65)	
< 0.75	172 (35)	123 (35)	49 (34)	
> 1.25	4 (1)	3 (1)	1 (1)	
Calcium (mmol/L)				0.929
2.25–2.75	29 (6)	20 (6)	9 (6)	
< 2.25	460 (93)	327 (94)	133 (93)	
> 2.25	3 (1)	2 (1)	1 (1)	
White blood cell (∗10^−9^/L)				0.029
4–10	152 (31)	103 (30)	49 (34)	
< 4	41 (8)	23 (7)	18 (13)	
> 10	299 (61)	223 (64)	76 (53)	
Hemoglobin (∗10^−9^/L)				0.97
110–160	256 (52)	181 (52)	75 (52)	
< 110	223 (45)	158 (45)	65 (45)	
> 160	13 (3)	10 (3)	3 (2)	
Platelet (∗10^−9^/L)				0.039
100–300	330 (67)	246 (70)	84 (59)	
< 100	138 (28)	87 (25)	51 (36)	
> 300	24 (5)	16 (5)	8 (6)	
Albumin (g/L)	28.6 ± 4.96	29.23 ± 5.07	27.06 ± 4.34	< 0.001
Globulin (g/L)	26.6 (23.48, 30.52)	27.3 (24, 31.4)	25 (22.2, 28)	< 0.001
SPO_2_ (%)	97 (95, 98.25)	98 (96, 99)	95 (92, 98)	< 0.001
Temperature (°C)				0.545
36–37.5	256 (52)	187 (54)	69 (48)	
< 36	27 (5)	18 (5)	9 (6)	
> 37.5	209 (42)	144 (41)	65 (45)	
MAP (mmHg)				0.929
70–105	289 (59)	206 (59)	83 (58)	
< 70	142 (29)	101 (29)	41 (29)	
> 105	61 (12)	42 (12)	19 (13)	
Heart rate (times/min)	99 (86, 117)	98 (85, 116)	105 (88, 120)	0.072
Breathe rate (times/min)	20 (18, 22)	20 (18, 20)	20 (20, 24)	< 0.001
GCS	15 (15, 15)	15 (15, 15)	15 (15, 15)	0.11
Diabetes				1
No	384 (78)	272 (78)	112 (78)	
Yes	108 (22)	77 (22)	31 (22)	
Hypertension				0.38
No	248 (50)	171 (49)	77 (54)	
Yes	244 (50)	178 (51)	66 (46)	
Cerebral infarction				1
No	469 (95)	333 (95)	136 (95)	
Yes	23 (5)	16 (5)	7 (5)	
Cancer				0.394
No	415 (84)	298 (85)	117 (82)	
Yes	77 (16)	51 (15)	26 (18)	
Chronic lung disease				0.887
No	474 (96)	337 (97)	137 (96)	
Yes	18 (4)	12 (3)	6 (4)	
Chronic heart disease				0.052
No	475 (97)	341 (98)	134 (94)	
Yes	17 (3)	8 (2)	9 (6)	
Chronic liver disease				0.701
No	469 (95)	334 (96)	135 (94)	
Yes	23 (5)	15 (4)	8 (6)	
Chronic kidney disease				0.999
No	449 (91)	319 (91)	130 (91)	
Yes	43 (9)	30 (9)	13 (9)	
Leukemia				1
No	488 (99)	346 (99)	142 (99)	
Yes	4 (1)	3 (1)	1 (1)	
Intracranial infection				1
No	490 (100)	347 (99)	143 (100)	
Yes	2 (0)	2 (1)	0 (0)	
Lung infection				< 0.001
No	348 (71)	271 (78)	77 (54)	
Yes	144 (29)	78 (22)	66 (46)	
Biliary infection				0.866
No	454 (92)	323 (93)	131 (92)	
Yes	38 (8)	26 (7)	12 (8)	
Urinary infection				0.434
No	397 (81)	278 (80)	119 (83)	
Yes	95 (19)	71 (20)	24 (17)	
Gastrointestinal infection				0.434
No	397 (81)	278 (80)	119 (83)	
Yes	95 (19)	71 (20)	24 (17)	

*Note:* SPO_2_, pulse oxygen saturation.

Abbreviations: GCS, Glasgow coma score; HS-CRP, high-sensitivity C-reactive protein; MAP, mean arterial pressure; pro-BNP, pro-brain natriuretic peptide.

^a^Continuous variables are described as means and quarterbacks due to non-normal distribution. Categories varies are analyzed by the *χ*^2^ test and continuous variables are analyzed by the Wilcoxon rank sum test.

^b^First examination index following admission.

**Table 3:** Multivariate logistic regression analysis and stepwise regression analysis of involved variables in the modeling group.

Variables	Multivariable logistic regression		Stepwise regression	
	OR (95% CI)	*p* value	OR (95% CI)	*p* value
Lactic acid (mol/L)	1.154 (1.060–1.264)	0.001	1.154 (1.060–1.263)	0.001
Pro-BNP^a^ (pg/mL)	1.000 (1.000–1.000)	0.007	1.000 (1.000–1.000)	0.007
Prothrombin time (s)	1.052 (0.993–1.112)	0.088	1.052 (0.993–1.117)	0.088
D-dimer (mg/L)	1.052 (1.011–1.094)	0.011	1.052 (1.011–1.094)	0.011
Albumin (g/L)	0.927 (0.879–0.976)	0.005	0.927 (0.879–0.976)	0.005
Globulin (g/L)	0.939 (0.901–0.975)	0.001	0.939 (0.901–0.975)	0.002
SPO_2_ (%)	0.811 (0.745–0.882)	< 0.001	0.812 (0.745–0.882)	< 0.001
Breathe rate (times/min)	1.000 (0.972–1.029)	0.985	NA	NA
Lung infection	2.505 (1.528–4.115)	< 0.001	2.505 (1.529–4.115)	< 0.001

^a^The exact pro-BNP in the multivariable logistic regression was 1.000031 (1.000008–1.000053); the exact value for pro-BNP in the stepwise regression was 1.00003 (1.000008–1.00005); SPO_2_, pulse oxygen saturation.

**Table 4:** The comparison of model performances based on different methods.

Models	AUC	Accuracy	Sensitivity	Specificity	PPV	NPV	Adjusted *p*^a^
Nomogram	0.795 (0.723–0.860)	0.786 (0.784–0.787)	0.689 (0.574–0.803)	0.826 (0.765–0.886)	0.618 (0.502–0.733)	0.866 (0.810–0.920)	Reference
SOFA	0.628 (0.544–0.713)	0.686 (0.448–0.767)	0.460 (0.148–0.918)	0.765 (0.262–0.987)	0.456 (0.329–0.875)	0.775 (0.736–0.894)	0.006
NEWS	0.618 (0.535–0.701)	0.581 (0.505–0.733)	0.705 (0.246–0.836)	0.524 (0.423–0.913)	0.382 (0.330–0.577)	0.811 (0.743–0.879)	0.007
C5.0	0.757 (0.677–0.836)	0.786 (0.695–0.838)	0.672 (0.492–0.820)	0.832 (0.664–0.926)	0.621 (0.484–0.761)	0.858 (0.813–0.908)	1.000
SVM	0.781 (0.708–0.853)	0.752 (0.643–0.819)	0.738 (0.557–0.902)	0.758 (0.550–0.886)	0.553 (0.441–0.700)	0.878 (0.825–0.941)	1.000
XGBoost	0.759 (0.678–0.839)	0.786 (0.695–0.852)	0.639 (0.443–0.803)	0.846 (0.684–0.803)	0.603 (0.485–0.900)	0.853 (0.804–0.906)	1.000
Ensemble	0.778 (0.699–0.857)	0.795 (0.738–0.857)	0.689 (0.508–0.803)	0.839 (0.758–0.960)	0.640 (0.536–0.850)	0.868 (0.819–0.913)	1.000

Abbreviations: NPV, negative predictive value; PPV, positive predictive value.

^a^ *p* values between any two compared groups were adjusted by Bonferroni correction.

## Data Availability

Data supporting our conclusion have been included within the article.
